# 2333: Examination of barriers and facilitators to sexual healthcare access among adolescent Latinas in Alabama

**DOI:** 10.1017/cts.2017.262

**Published:** 2018-05-10

**Authors:** Mercedes Margarita Morales Aleman, Isabel C. Scarinci, Gwendolyn Ferreti

**Affiliations:** The University of Alabama, Tuscaloosa, AL, USA

## Abstract

OBJECTIVES/SPECIFIC AIMS: Alabama (AL) experienced a 145% increase in its Latino population between 2000 and 2010; making it the state with the second fastest growing Latino population in the United States (US) during that time. Adolescent Latinas in the US and in AL are disproportionately affected by sexual health disparities as evidenced by the disproportionate burden of HIV, STIs and early pregnancy compared with their non-Hispanic, White counterparts. Empirical data with adult Latinas in the southeast suggest significant barriers to sexual healthcare access. However, to our knowledge, no other researchers have examined barriers and facilitators to sexual healthcare access for this subpopulation. Therefore, the purpose of this study is to examine adolescent Latinas’ sexual healthcare needs through in-depth qualitative interviews. These qualitative interviews (phase 1 of a 3-phase study) will inform the development of community-driven, theory-based, culturally-relevant, multi-level intervention strategies to reduce sexual health disparities and increase sexual healthcare access for this group. Community-based participatory research (CBPR), which ensures equitable participation of stakeholder groups through partnerships, and the socioecological model of health, which conceptualizes the individual as nested within a set of social structures, provide the philosophical and theoretical frameworks for the work. METHODS/STUDY POPULATION: Between January and March of 2017, we will conduct 30 qualitative interviews with eligible adolescents who: self-identify as Latina, are between 15 and 19 years old, have been in the US for over 5 years, and live west AL. We will use venue-based, purposeful convenience sampling to recruit participants. We will manage and analyze the data with the qualitative software NVivo 10. We will use a multi-step, consensus-based process to code and analyze the interviews in the language in which they were conducted (ie, Spanish or English). We will maintain detailed audit trails during the analysis process and seek an inter-rater reliability of 0.85. RESULTS/ANTICIPATED RESULTS: We expect to identify barriers and facilitators to sexual healthcare services at distinct levels of the socioecological model of health. Study results and implications for practice in clinical settings will be discussed in detail. DISCUSSION/SIGNIFICANCE OF IMPACT: The proposed research is significant because (1) the state of AL experienced a dramatic increase in its Latino/a population over the last 15 years and adolescent Latinas in AL are disproportionately affected by sexual health disparities; (2) to our knowledge, this will be the first study to examine the multi-level factors associated with sexual healthcare access for adolescent Latinas in the South and inform intervention strategies to promote sexual healthcare access in this population; (3) the work will be conducted under the philosophical lens of CBPR such that community members will be involved in every step of the research process, resulting in culturally relevant intervention strategies.

